# Heterologous production and characterization of a pyomelanin of Antarctic *Pseudomonas* sp. ANT_H4: a metabolite protecting against UV and free radicals, interacting with iron from minerals and exhibiting priming properties toward plant hairy roots

**DOI:** 10.1186/s12934-022-01990-3

**Published:** 2022-12-16

**Authors:** Michal Styczynski, Agata Rogowska, Christine Nyabayo, Przemyslaw Decewicz, Filip Romaniuk, Cezary Pączkowski, Anna Szakiel, Roderich Suessmuth, Lukasz Dziewit

**Affiliations:** 1 Department of Environmental Microbiology and Biotechnology, Institute of Microbiology, Faculty of Biology, University of Warsaw, Warsaw, Poland; 2 Department of Plant Biochemistry, Institute of Biochemistry, Faculty of Biology, University of Warsaw, Warsaw, Poland; 3 Institute of Chemistry, Technical University of Berlin, Berlin, Germany

**Keywords:** Antarctica, Biopolymer, *Pseudomonas*, Priming of hairy roots, Pyomelanin

## Abstract

**Background:**

Antarctica has one of the most extreme environments in the world. This region is inhabited by specifically adapted microorganisms that produce various unique secondary metabolites (e.g. pigments) enabling their survival under the harsh environmental conditions. It was already shown that these natural, biologically active molecules may find application in various fields of biotechnology.

**Results:**

In this study, a cold-active brown-pigment-producing *Pseudomonas* sp. ANT_H4 strain was characterized. In-depth genomic analysis combined with the application of a fosmid expression system revealed two different pathways of melanin-like compounds biosynthesis by the ANT_H4 strain. The chromatographic behavior and Fourier-transform infrared spectroscopic analyses allowed for the identification of the extracted melanin-like compound as a pyomelanin. Furthermore, optimization of the production and thorough functional analyses of the pyomelanin were performed to test its usability in biotechnology. It was confirmed that ANT_H4-derived pyomelanin increases the sun protection factor, enables scavenging of free radicals, and interacts with the iron from minerals. Moreover, it was shown for the first time that pyomelanin exhibits priming properties toward *Calendula officinalis* hairy roots in in vitro cultures.

**Conclusions:**

Results of the study indicate the significant biotechnological potential of ANT_H4-derived pyomelanin and open opportunities for future applications. Taking into account protective features of analyzed pyomelanin it may be potentially used in medical biotechnology and cosmetology. Especially interesting was showing that pyomelanin exhibits priming properties toward hairy roots, which creates a perspective for its usage for the development of novel and sustainable agrotechnical solutions.

**Supplementary Information:**

The online version contains supplementary material available at 10.1186/s12934-022-01990-3.

## Background

Antarctica is one of the most extreme regions on Earth, characterized by very low temperatures, strong ultra-violet (UV) radiation, and sunlight and nutrient deficiency [1]. However, even harsh polar environments are inhabited by highly specialized microorganisms that have developed a number of adaptive traits necessary for their survival [2]. These include the synthesis of various secondary metabolites, e.g., biosurfactants and siderophores, which help to increase the availability of nutrients and minerals [3] or the synthesis of pigments (carotenoids and melanins) responsible for protection against UV radiation [4, 5].

An important group of microorganisms often isolated from the Antarctic regions is bacteria of the genus *Pseudomonas* [6]. These rod-shaped, Gram-negative bacteria are valued in biotechnology due to their high metabolic potential for the production of several valuable secondary metabolites [7]. Among many known compounds, pseudomonads produce bioactive pigments classified as phenazines (pyocyanin and pyorubrin), siderophores (pyoverdine, fluorescein), and melanins (pyomelanin) [8]. While much is known about the basic functions of the above-mentioned secondary metabolites, the potential of melanins is still not fully exploited.

Melanins are brown-black polymers produced by organisms mainly to protect against UV radiation [9]. These bioactive polymers are classified according to the chemical precursors used in their biosynthesis, being eumelanin, pheomelanin, neuromelanin, allomelanin, and pyomelanin, the latter of which is also classified as a form of allomelanin [10, 11]. Pyomelanin originates from the catabolism of L-tyrosine or L-phenylalanine which, due to further transformation by 4-hydroxyphenylpyruvate dioxygenase (Hpd), forms homogentisic acid (HGA). HGA is the substrate of homogentisate 1,2-dioxygenase (HmgA). However, overexpression of Hpd or repression of HmgA leads to accumulation of HGA and causes its secretion from the cell where it undergoes oxidation to benzoquinoneacetic acid and self-polymerization into pyomelanin [10, 12]. Moreover, due to the heterogenous nature of the polymer [13] and under certain circumstances, additional substrates may appear during its formation as a consequence of, e.g., quinone oxidoreductases activity [14–16].

The chemical structure of pyomelanin is responsible for numerous biological properties and functions such as protection from light and oxidative stress, energy transduction as well as chelation and reduction of metal ions, e.g., Fe^3+^ [10, 17]. It has been suggested that pyomelanins may contribute to increasing the availability of certain elements in minerals for the microorganisms [9, 10, 17]. They can, therefore, play a supporting role for bacterial siderophores through increasing iron bioavailability [18]. On the other hand, since melanins produced by certain plant pathogens are virulence factors [19], these compounds, as elicitors, can stimulate defense responses in plants [20]. Checking whether a certain compound can be used as a potential elicitation agent very often involves examining its effect on the production of general and specialized metabolites in native plants or their in vitro cultures. The results are often manifested in the modified proportions of both types of metabolites [21]. In summary, these data confirm that melanins have considerable biotechnological potential.

In this study, the identification and functional characterization of pyomelanin originating from the Antarctic, psychrotolerant *Pseudomonas* sp. ANT_H4 was performed. The subsequent optimization of the pyomelanin production allowed revealing biological functions of this pigment as well as its potential for being used in environmental biotechnology.

## Results and discussion

### General physiological characterization of *Pseudomonas* sp. ANT_H4

*Pseudomonas* sp. ANT_H4 shows growth at temperatures from 4 to 37 °C (optimum 18 °C), tolerates a pH of 6–10, and can survive in NaCl concentrations of up to 3% [6]. It is also worth mentioning that ANT_H4 exhibits many features considered to be environmental adaptation strategies: ANT_H4 is able to produce siderophores in amounts of ~ 0.7 mmol/l as well as biosurfactants, which are able to lower the interfacial tension of a medium up to ~ 55% (from 55 (± 2) to 30 (2 ±) mN/m). An important feature distinguishing ANT_H4 from most of the other Antarctic *Pseudomonas* strains studied is its ability to sequester a brown-black pigment into the medium, in amounts of ~ 0.01 mg/mL.

### Overproduction of melanin-like compounds from fosmid clones

**Fig. 1:**
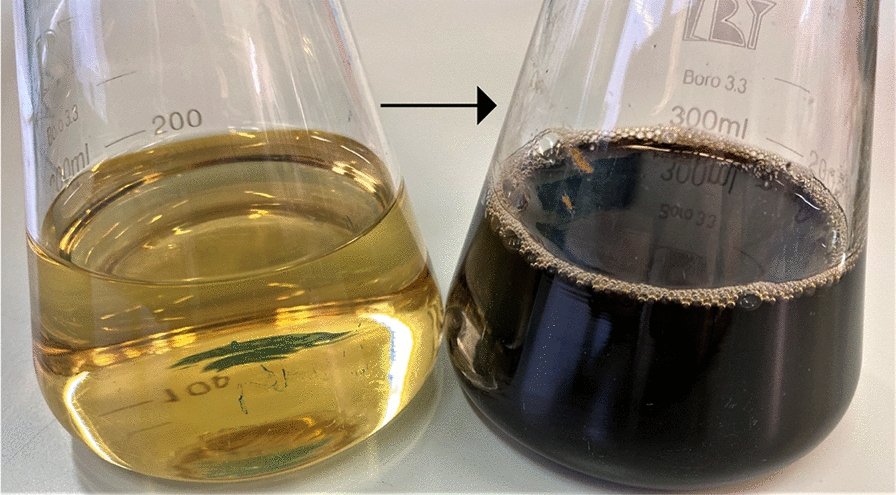
Overproduction of melanin-like compound in *E. coli* MPO554 carrying fosmid with appropriate ANT_H4 genome fragment. The left flask shows the uninoculated LB medium, while the right flask presents the ANT_H4 culture cultivated for 120 h using the same medium

From among more than 3000 *E. coli* MPO554 fosmid clones carrying ~ 45 kb genome fragments of *Pseudomonas* sp. ANT_H4 genome, we selected those that produced brown-black pigment into the medium. The expression medium supplemented with L-tyrosine allowed fosmid clones to efficient production of a brown-black pigment in amount of 0.25 mg/mL (Fig. 1). The obtained metabolite was compared chromatographically to that obtained from the native strain ANT_H4 and revealed identical nature. This is the first time that an overproduction of a melanin-like compound has been achieved through a fosmid expression system. The single-gene heterologous expression systems constructed by other scientists enabled to receive ~ 0.213 mg/mL from *E. coli* JM109 [22] and ~ 0.315 mg/mL from *E. coil* KSYH [23] using the 4‑hydroxyphenylpiruvate dioxygenase gene from *P. aeruginosa* and *Ralstonia pickettii*, respectively.

### Identification of a melanin-like compound produced by *Pseudomonas* sp. ANT_H4

As mentioned above *Pseudomonas* sp. ANT_H4 showed the unique ability to sequester a brown-black pigment into the medium. Based on the literature review, we concluded that this may be a melanin-like compound [24–26]. For its identification, HPLC-DAD and FT-IR analyses were performed.

**Fig. 2:**
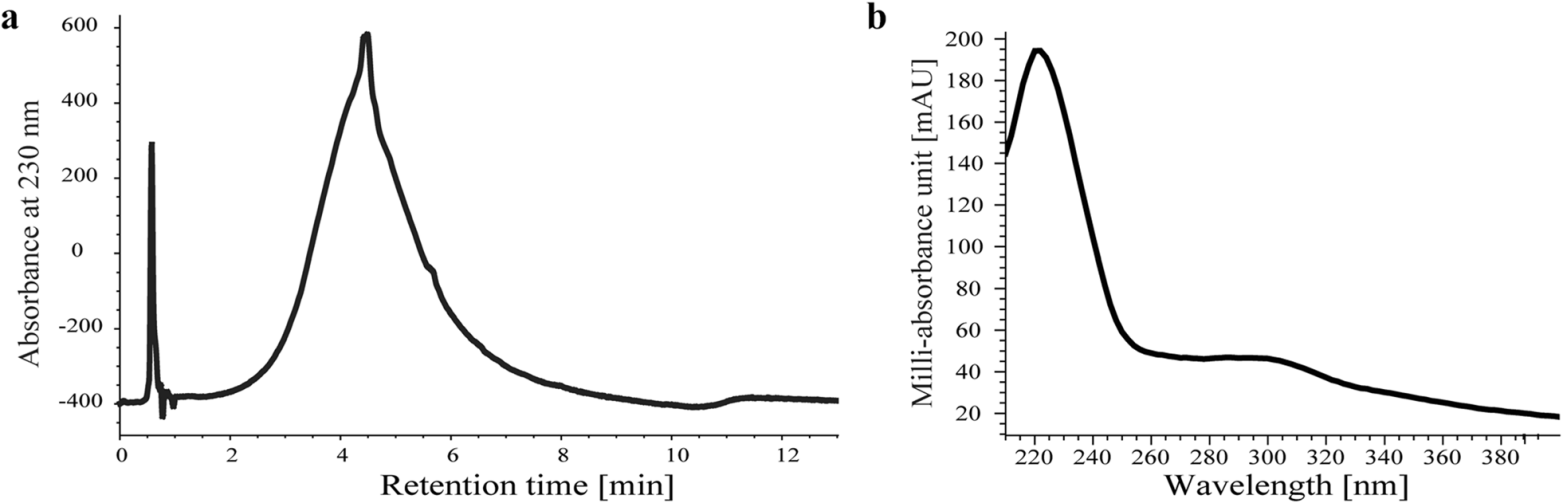
Analytical characterization of the ANT_H4-derived melanin-like compound. **a** reversed-phase C18 HPLC chromatogram, revealing a broad peak with a retention time Rt = 4.2 min, and **b** UV/Vis spectrum with absorption maxima at λ = 230 nm

Analysis by HPLC using a DAD detector allowed for the determination of three basic characteristics of the compound. First, the comparatively high polarity due to poly-quinone pyomelanin structure [10], as evidenced by a relatively short retention time (R_t_ = 4.2 min, by Fig. 2A) in the water/acetonitrile gradient. Second, the maximum UV absorbance of the compound at a wavelength of λ = 230 nm (Fig. 2B), which is a characteristic feature of compounds from the melanin group [27]. Three, a very broad peak typical for polymers [28].

**Fig. 3:**
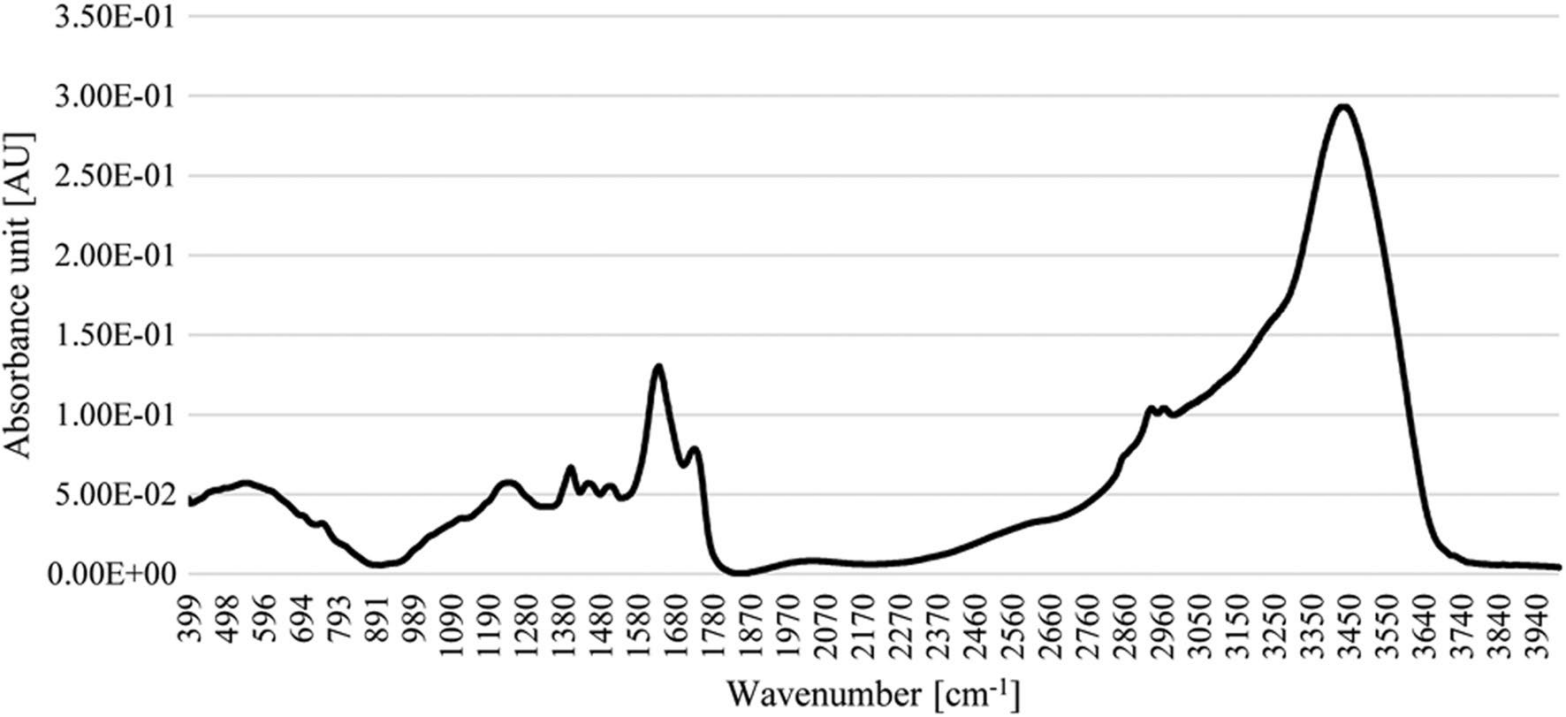
FTIR spectrum of a preparation of the ANT_H4-derived melanin-like metabolite

In addition, the FT-IR spectroscopy of a sample of the ANT_H4-derived melanin-like metabolite was performed. The analysis of the IR spectra revealed several melanin-characteristic signals [29–31], i.e., a broad band at 3434 cm^−1^ assigned to the presence of an OH group (tensile vibration), a band at 1728 cm^−1^ corresponding to vibrational excitation of carbonyl groups C = O or of COOH groups, a band at 1634 cm^−1^ may come from the ionized COO^−^ groups, at 1403 cm^−1^ indicating vibration from ionized COO^−^ groups, and at 1241 cm^−1^ that may be a signal from the combination of C-O bonds (Fig. 3).

Altogether, the performed HPLC and FTIR analyses confirmed the presence of a melanin-like compound classified as heterogenous pyomelanin [32], which is produced by some *Pseudomonas* species [24–26].

### Genomic characterization of *Pseudomonas* sp. ANT_H4 – tracking of genes responsible for pyomelanin production

Sequencing of the *Pseudomonas* sp. ANT_H4 genome using the Illumina MiSeq platform generated 1,030,406 paired reads and 319 Mbp of sequence information. As a result of the assembly of the *Pseudomonas* sp. ANT_H4 genome, 89 contigs of a total length of 6,131,973 bp were obtained. Initially, the genome sequence was automatically annotated using RAST on the PATRIC 3.6.2 web service and its general features for the ANT_H4 strain are presented in Table 1.

**Table 1:** General features of the *Pseudomonas* sp. ANT_H4 draft genome

Feature	Calculation
Strain	ANT_H4
Number of contigs	89
Estimated genome size (bp)	6,131,973
GC content (%)	58.6
Number of genes	6174
Number of proteins with functional assignments	4692
Number of proteins with EC number assignments	1267
Number of tRNA genes	59
Number of regulatory RNA genes	6

In-depth genomic analyses of the *Pseudomonas* sp. ANT_H4 strain revealed its considerable metabolic potential. Besides the set of genes belonging to the primary metabolism, e.g., glycolysis, gluconeogenesis, the citrate cycle, the pentose phosphate and Entner-Doudoroff pathways, the ANT_H4 strain possesses complete metabolic pathways of dissimilatory nitrate reduction and denitrification as well as a pathway of assimilatory sulfate reduction. These findings suggest it has high adaptation to surviving under anaerobic conditions, which is also characteristic of many other known pseudomonads [33–35]. Moreover, in the ANT_H4 genome, there are genes encoding enzymes responsible for biosynthesis and utilization of polyhydroxyalkanoates (i.e., polyhydroxyalkanoic acid synthases (EC:2.3.1.-) (GenBank accession numbers: KAA0949046, KAA0949048) and poly (3-hydroxyalkanoate) depolymerase (EC:3.1.1.75) (GenBank accession number: KAA0949047)), as well as enzymes responsible for the production of surface-active rhamnolipids (i.e., RhlA) (GenBank accession number: KAA0950251), RmlABCD (GenBank accession numbers: KAA0945161, KAA0943063, KAA0948219, KAA0945160, KAA0945162, and KAA0945163) – metabolites crucial for survival under nutrient deficiency, permanent cold, and freezing conditions [36, 37]. Furthermore, in the ANT_H4 genome there are also gene clusters encoding for the synthesis of two types of siderophores, being xanthoferrin – PvsABCDE (GenBank accession numbers: KAA0945766, FQ182_15965 (locus; frameshifted), KAA0945767, KAA0945768, KAA0945769) and pyoverdine –PvdAEHLMOPQSY (GenBank accession numbers: KAA0947683, KAA0947675, KAA0944452, KAA0944446, KAA0947677, KAA0947676, KAA0947679, KAA0945967, KAA0944444, and KAA0944443), necessary in nutrient-deficient environments and acting as iron chelators.

As mentioned above, *Pseudomonas* sp. ANT_H4 produces a brown-black pigment that was identified as pyomelanin. Based on the genomic analysis, it was shown that ANT_H4 possesses a known and common pathway of tyrosine degradation to homogentisic acid, meaning it encodes tyrosine aminotransferase (EC:2.6.1.5) (GenBank accession number: KAA0943952) and 4-hydroxyphenylpyruvate dioxygenase (EC:1.13.11.27) (GenBank accession number: KAA0943679). The homogentisic acid may be then used as a starting material for pyomelanin production. However, in the genome, an enzyme involved in homogentisic acid degradation (homogentisate 1,2-dioxygenase (EC:1.13.11.5) (GenBank accession number: KAA0946993) is also encoded. Nevertheless, the presence of quinone oxidoreductases (EC:1.6.5.5) (GenBank accession numbers: KAA0947416, KAA0946026, KAA0945409), which detoxify benzoquinones by reducing them into hydroquinones, may interrupt proper homogentisic acid degradation, which manifests in pyomelanin production [38–40].

**Fig. 4:**
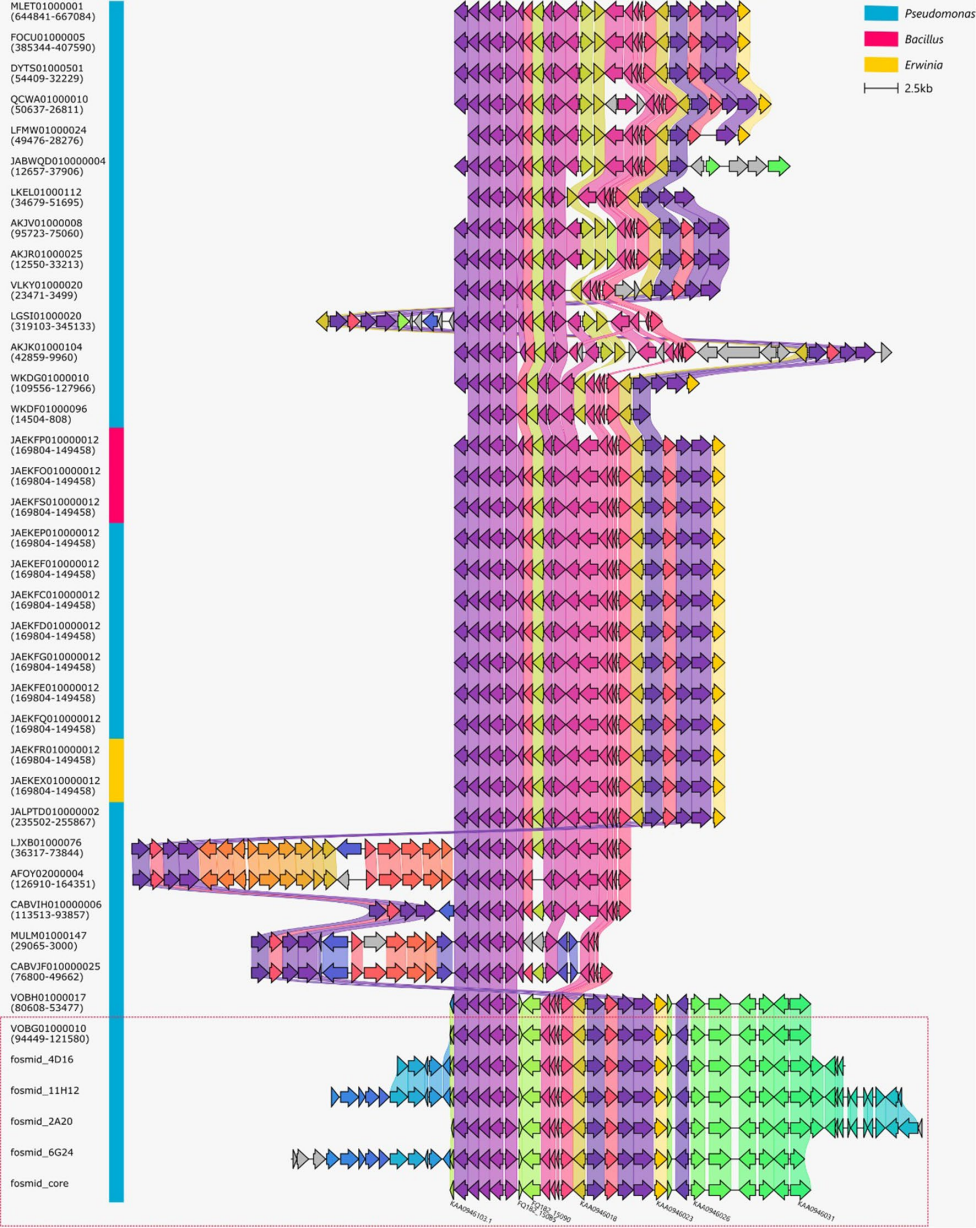
A comparison of the fosmid-encoded core proteome with other bacterial genomes. The latter were selected based on the presence of at least 10 homologous proteins encoded within a 50-kb-range of DNA sequence. From these, only homologous regions were extracted based on coordinates present in brackets below the accession number of a particular contig. They were aligned with a clinker tool, where each protein coding sequence is represented as a rectangle arrow. Homologous proteins have the same color and proteins from adjacent contigs are connected with a block of a lighter equivalent of their color. The genus of the host bacterial strain is color-coded next to accession numbers. Fragments originating from the genome of ANT_H4 were marked with a dashed, red frame. Additionally, for the sake of figure clarity, only selected proteins from the fosmid-encoded core proteome were labeled with an accession number or locus_tag

To identify genes directly responsible for the production of pyomelanin, functional analysis using a fosmid-based expression system was applied [41]. Four fosmid clones displaying the capacity of synthesizing pyomelanin were further sequenced. The analysis of these sequencing results revealed a common core set of 27 genes (Fig. 4, Table 2). Amongst the identified enzymes, we found one gene function that might be directly involved in the melanin-overproducing phenotype: quinone oxidoreductase (EC 1.6.5.5). The gene encoding for this enzyme was amplified by PCR, subcloned into plasmid pBluescript II SK, and transformed into *E. coli* TG1 and MPO554. However, the pyomelanin production phenotype was not observed in any of the tested hosts, which may indicate that either the introduced gene is inactive, not involved in pyomelanin production, or not sufficient for pyomelanin overproduction. Taking into account that all fosmids in pyomelanin-producing strains contained a set of common genes we assume that this is not a single-gene-generated phenotype, but rather a consequence of the action of several genetic determinants creating a unique cluster.

**Table 2:** Shared proteins (core proteome; fosmid_core) of four sequenced ANT_H4-derived fosmids responsible for pyomelanin formation by heterologous *E. coli* host

GenBank accession number	Predicted function
KAA0946031	RimK family protein
KAA0946030	N-acetyltransferase (GNAT family) (EC 2.3.1.-)
KAA0946029	Dimethyl sulfone monooxygenase (EC 1.14.13.131)
KAA0946028	Acyl-CoA dehydrogenase (EC 1.3.8.-)
KAA0946027	Phosphoglucomutase (EC 5.4.2.2)
KAA0946026	Quinone oxidoreductase (EC 1.6.5.5)
KAA0946025	Transcriptional regulator MexT
KAA0946024	Hypothetical protein
KAA0946023	Protein involved in meta-pathway of phenol degradation
KAA0946022	Aldehyde dehydrogenase (EC 1.2.1.3)
KAA0946021	NAD(P)-dependent alcohol dehydrogenase (EC 1.1.1.71)
KAA0946020	Catechol 1,2-dioxygenase (EC 1.13.11.1)
KAA0946019	Salicylate 1-monooxygenase (EC 1.14.13.1)
KAA0946018	Transcriptional regulator (LysR family)
KAA0946017	Transcriptional regulator (LysR family)
KAA0946016	4-oxalocrotonate tautomerase family protein
KAA0946104	hypothetical protein
KAA0946015	Nitroreductase
FQ182_15090	Uncharacterized MFS-type transporter
FQ182_15085	Oxidoreductase (short-chain dehydrogenase/reductase family, frameshifted)
KAA0946014	Transcriptional regulator (LysR family)
KAA0946013	Luciferase-like monooxygenase (LLM) class oxidoreductase
KAA0946012	Xylose isomerase domain (TIM barrel protein)
KAA0946011	Xylose isomerase domain (TIM barrel protein)
KAA0946010	LLM class flavin-dependent oxidoreductase
KAA0946103	Transcriptional regulator (ArsR family)

We further explored the presence and co-location of genes encoding for proteins homologous to proteins encoded by the core of fosmids giving the desired phenotype. After a search of the NCBI nr database, we observed that there were 64 contigs carrying at least 10 out of 23 homologous genes and 34 of them carried these genes within a 50-kb-range fragment of DNA (Fig. 4). Besides the ANT_H4 contig (accession number VOBG01000010), we identified the same gene cluster in another strain isolated from the same location – *Pseudomonas* sp. ANT_H12B (VOBH01000017). Other strains encoded at most 15 homologous proteins. It is also worth noting that within the set of genomic fragments, only *P. lurida* PGSB 7828 (D010000004) encoded for a quinone oxidoreductase homolog.

### Evaluating the biotechnological potential of the identified pyomelanin

#### Sun-protection and free radical scavenging activity

One of the basic functions of the compounds from the melanin group is protection against UV radiation. The ability of ANT_H4-derived pyomelanin to absorb UV light contributes to direct protection from sunlight. As a non-cytotoxic compound [15, 25, 42], pyomelanin could be an excellent additive to enhance the power of sunscreens. In order to test that hypothesis, the sun protection factor (SPF) of three commercial sunscreens was determined empirically: The stated SPFs were 5, 15, and 25, while the results of the analyses revealed values of 5.09 ± 0.03, 13.41 ± 0.35, and 25.10 ± 0.13, respectively. Addition of pyomelanin to each of these sunscreens resulted in an increase of SPF to: (i) 7.79 ± 0.04, 15.88 ± 0.43, 27.48 ± 0.12 when pyomelanin in final concentration of 0.1 mg/mL was added, and (ii) 6.41 ± 0.14, 14.65 ± 0.19, 25.35 ± 0.17 when pyomelanin in final concentration of 0.01 mg/mL was added [25, 43, 44]. The effect of ANT_H4-derived pyomelanin is consistent with the results of other researchers testing the SPF changes of sunscreen [25, 42, 45]. However, currently, only two melanin-like compounds are used in cosmetics; catechol melanin from black olive fruits (Laboratoires Biocyte, France), and "bio-melanin" in anti-UV lotions (Baar Products, PA, USA). So far, there is no mention of the use of pyomelanin, mainly due to the lack of optimization of its production efficiency [42].

**Fig. 5:**
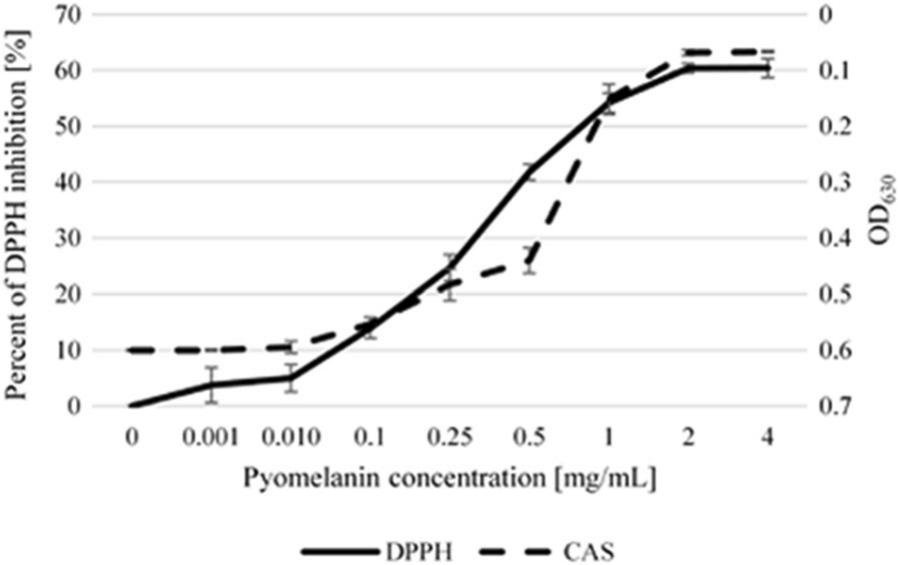
The ability of ANT_H4-derived pyomelanin to scavenge DPPH free radicals and chelate iron CAS reagent. Error bars represent the standard deviations of the triplicates

UV radiation (especially UVA) reaching the skin leads to the generation of free radicals that oxidize proteins, lipids, and nucleic acids [46]. In the case of pyomelanin, the ability to scavenge free radicals is its primary feature [24, 47]. In order to test the antioxidant capacity of the ANT_H4-derived pyomelanin, the free-radical scavenging effect was tested on a 0.1 mM DPPH solution (used as a free radical). The IC_50_ value (antioxidant concentration required for quenching 50% of the initial DPPH) was established at a concentration of ~ 0.75 mg/mL pyomelanin, which quenched maximally 60% of the DPPH at a concentration of 2 mg/mL (Fig. 5). This is in line with the results of previous studies [15, 17, 45].

It is worth mentioning that the antioxidant activity significantly increases the applicability of the tested compound in cosmetology. This feature is highly desirable in, e.g., creams or lotions that reduce the negative impact of free radicals on skin aging.

#### Iron binding properties

One of the features of pyomelanin is its ability to interact with iron-containing particles. The presence of quinones in the polymer structure, as well as the specific structure resembling siderophores in certain configurations, enables both the binding and reduction (Fe^3+^ to Fe^2+^) of iron [10, 48]. For example, pyomelanin produced by *Legionella pneumophila* conferred a ferric reductase activity and thus played an important role in iron uptake [18, 30, 49]. This is a feature that leads to an increase in iron bioavailability and thus positively impacts bacterial fitness. The iron-binding properties of ANT_H4-derived pyomelanin were performed against the CAS-HDTMA-iron complex, in which iron is relatively weakly bonded [50]. The iron chelation capacity of pyomelanin was measured spectrophotometrically at OD_630_. The results were similar to deferoxamine mesylate siderophore at a concentration of 0.025 mM and reached its maximum at a concentration of 2 mg/mL. Therefore, it suggests the participation of the tested compound in increasing the availability of iron in the environment, especially as the structure of the pyomelanin and its iron binding mechanism are similar to humic acids, commonly used in bio-fertilizers for plants [51–54].

Many iron chelators also exhibit antioxidant properties [55]. It was shown that the iron-binding and free-radical scavenging properties of ANT_H4-derived pyomelanin correlated and reached their maximum at a concentration of 2 mg/mL of pyomelanin (Fig. 5). This result is very important according to the iron-reduction properties of the investigated compound, which can lead to Fenton's reaction, responsible for the generation of free radicals [54, 56].

#### Bioconsolidation with ANT_H4-derived pyomelanin

Considering the pyomelanin iron-binding properties, its influence on minerals was tested. Bioconsolidation studies were conducted with the use of pyrite and hematite. Their basic composition is FeS_2_ and Fe_2_O_3_, i.e., the iron is in a reduced (Fe^2+^) and oxidized (Fe^3+^) form, respectively.

The batching experiment revealed increased iron retention in the presence of ANT_H4-derived pyomelanin. Both tested concentrations, i.e., 0.1 and 0.01 mg/mL significantly reduced the loss of iron after 1, 24, and 48 h of batching minerals. The results after 48 h were similar for both pyomelanin concentrations and showed over 80-fold increased hematite iron retention compared to the control. In the case of pyrite, an increased (over 60-fold) retention was observed with the use of a higher (0.1 mg/mL) concentration of pyomelanin (Table 3). The results are very promising in light of ANT_H4-derived pyomelanin applicability. Due to the specific chemical structure of pyomelanin, it participates in iron redox processes, but we also hypothesize that the polymer may adhere to the surface of minerals and stabilize them by creating a protective layer.

**Table 3:** The amount of iron released (µg/mL) from hematite and pyrite after treatment with pyomelanin at two concentrations and deionized water (ddH_2_O; control)

Mineral	Time [h]	Control		Pyomelanin			
		ddH_2_O	±	concentration 0.1 mg/mL	±	concentration 0.01 mg/mL	±
Hematite (Fe_2_O_3_)	1	1.878	0.087	0.042	0.005	0.044	0.001
	24	1.458	0.036	0.028	0.003	0.030	0.002
	48	1.386	0.029	0.016	0.005	0.016	0.004
Pyrite (FeS_2_)	1	1.174	0.134	0.050	0.005	0.049	0.000
	24	0.824	0.067	0.029	0.008	0.022	0.008
	48	0.571	0.038	0.009	0.004	0.016	0.003

#### Hairy roots elicitation

Melanin-like compounds are often identified among many fungal plant pathogens as their virulence factors protecting non-specifically against host defense mechanisms. The pathogen-free molecules themselves are not harmful to plants, however, they are recognized by them as a potential threat [47]. This initiates a cascade of biochemical processes leading to modifications of biosynthetic pathways resulting in different proportions of plant general and specialized metabolites, and thus to a preventive enhancement of the systemic defense response [21].

Sterols are considered to be plant general metabolites as they build cell membranes and are in charge of maintaining the fluidity and permeability of these structures. Phytosterols also play a role in adaptation to many stress (mostly abiotic) conditions [57]. Pentacyclic triterpenoids are plant specialized metabolites that take part in defense strategies against herbivores and pathogens [58]. During stressful conditions as well as upon elicitation, biosynthesis pathways of sterols and pentacyclic triterpenoids may be competitive due to the common precursor of both groups of compounds, i.e., squalene [21]. However, some factors may stimulate both pathways in parallel, showing evidence that plants’ defense strategies are combined and factor-dependent.

**Fig. 6:**
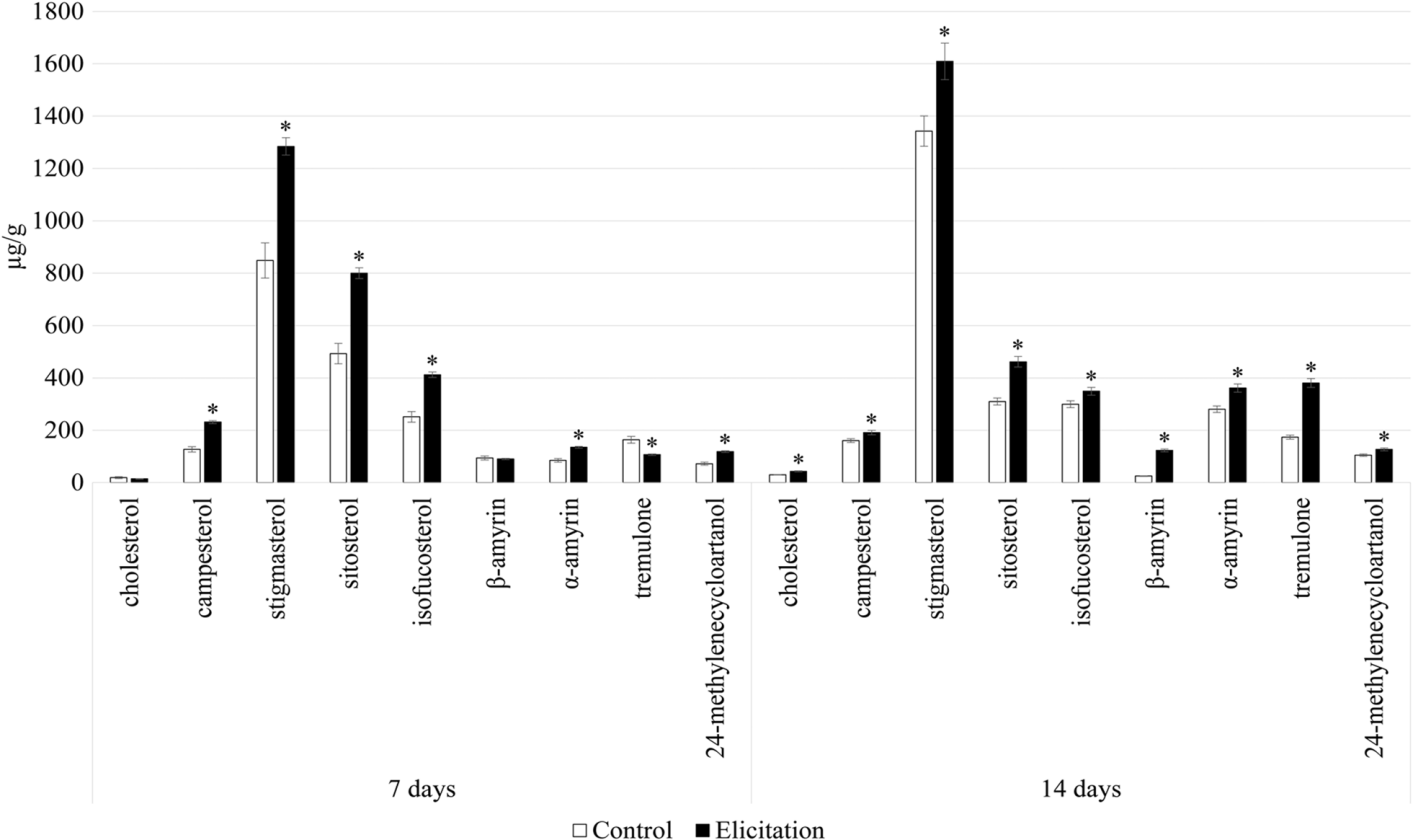
The content of steroids and triterpenoid alcohols in hairy root tissue. Error bars represent standard deviations of the triplicates. *—mean statistical significance p < 0.05

The sterol fraction obtained from diethyl ether extracts was composed of a group of typical sterols, namely campesterol, cholesterol, isofucosterol, sitosterol, and the predominating stigmasterol. One steroid ketone, tremulone, and one biosynthetic precursor, 24-methylenecycloartanol, were also identified in this fraction (Additional file 1: Table S1). The presence of pyomelanin in the culture medium caused a significant increase in steroid biosynthesis; the total content of these compounds in pyomelanin-elicited hairy roots was approximately 58% and 24% higher than in the control roots after 7 and 14 days, respectively (Fig. 6, Additional file 1: Table S2).

**Fig. 7:**
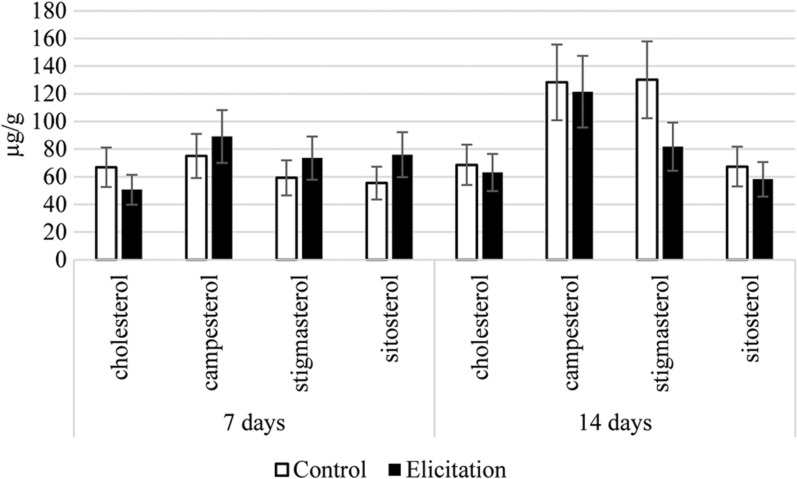
The content of sterol glycosides in hairy root tissue. Error bars represent standard deviations of the triplicates. *—mean statistical significance p < 0.05

Four sterols, i.e., cholesterol, campesterol, sitosterol, and stigmasterol, were also found in glycosides of methanol extracts subjected to hydrolysis (Fig. 7, Additional file 1: Table S3). The contents of sterol glycosides were similar in the control and elicited samples. After seven days of incubation, the number of sterols slightly increased (13%) in the elicited samples, however, after 14 days it decreased (22%) in comparison with control. The modifications in the ratio of conjugated to free forms of sterols are often considered a major key to modulating the membrane order and membrane biophysical properties. The changes in the content of sterol glycosides are often observed during plant stress reactions due to their influence on such plant membrane properties as fluidity and permeability. These changes can also influence the activity of some membrane-bound enzymes as well as proton pumps, which can lead to further modifications of various metabolic pathways [59].

**Fig. 8:**
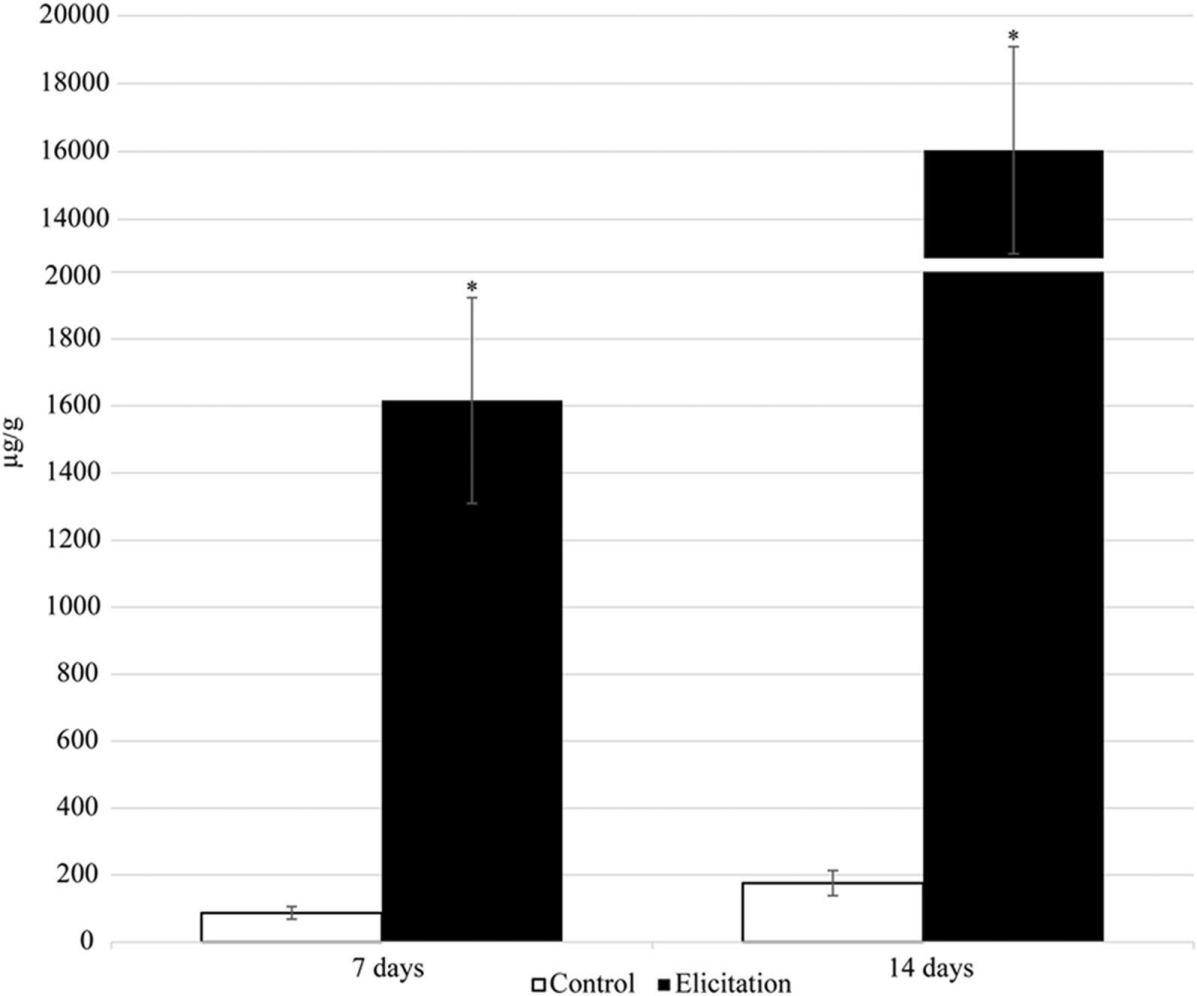
Free oleanolic acid produced by hairy root tissue. Error bars represent standard deviations of the triplicates. *—mean statistical significance p < 0.05

**Fig. 9:**
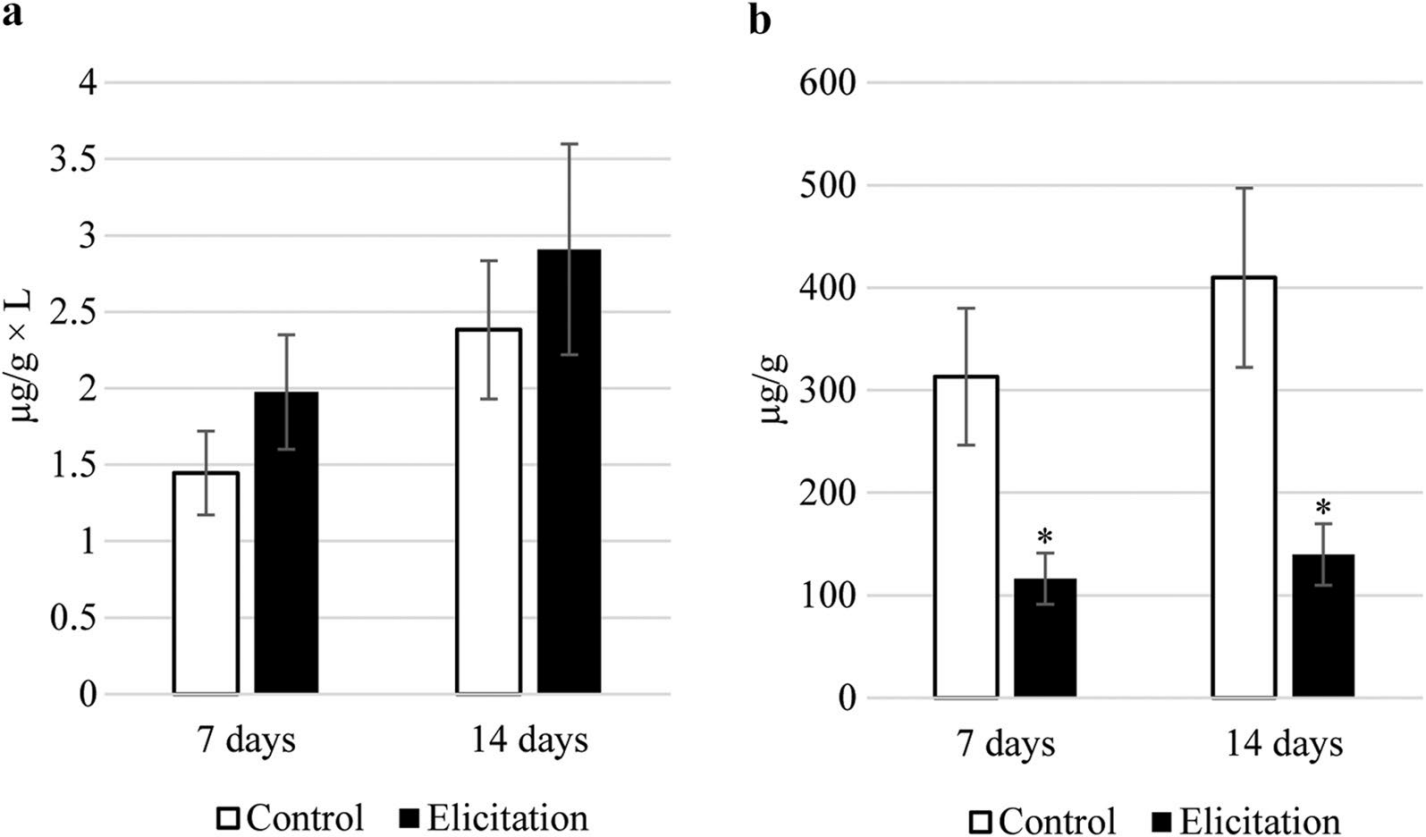
Oleanolic acid saponins released to the culture medium (a) and produced by hairy root tissue (b). Error bars represent standard deviations of the triplicates. *—mean statistical significance p < 0.05

A notable effect of pyomelanin elicitation was observed in the content of free oleanolic acid in hairy roots tissue – it increased more than 18-fold after 7 days and more than 90-fold after 14 days in the elicited samples (Fig. 8, Additional file 1: Table S4). Simultaneously, the content of the oleanolic acid precursor, triterpenoid alcohol β-amyrin, increased almost tenfold after 14 days of elicitation (Additional file 1: Table S2). The number of oleanolic acid saponins released into the medium was also higher in pyomelanin-treated samples, but the differences were not statistically significant (Fig. 9A, Additional file 1: Table S5). At the same time, the content of oleanolic acid saponins accumulated in the hairy roots tissue of the elicited samples was around 2.7- and 2.9-fold lower as compared with the control after 7 and 14 days, respectively (Fig. 9B, Additional file 1: Table S6). This might be explained by the increased release to the medium, however, the observed enhanced saponin secretion does not fully compensate for the decrease in their accumulation in root tissue occurring after elicitation. Therefore, it seems that it is the free oleanolic acid, and not its saponins, that plays the main role in *Calendula officinalis* Linnaeus hairy roots defense strategy against potential virulence factors.

The diverse effects of several elicitors including chitosan, heavy metals, and phytohormones, on the biosynthesis of sterols and triterpenoids in *Calendula officinalis* L. hairy roots, have been reported previously [59, 60]. The most spectacular effect observed so far was the 113-fold increase of oleanolic acid saponin release to the medium after elicitation with jasmonic acid [61]. However, such a high boost to the synthesis of free oleanolic acid as demonstrated in the present study (a more than 90-fold increase after elicitation with ANT_H4-derived pyomelanin) is observed for the first time and has never been reported previously. This result indicates that ANT_H4-derived pyomelanin elicitation is particularly effective in the stimulation of biosynthesis of triterpenoids occurring in a free, and not glycosidic, form. Such specificity in the modulation of the triterpenoid biosynthetic pathway seems to be a rather unique feature of the applied elicitor, and it might be of particular importance for the potential enhancement of biosynthesis of other bioactive triterpenoids.

The obtained results point to the possibility of the application of received pyomelanin in agrotechnics, e.g., as a potential plant priming agent. However, the use of this type of elicitor still requires additional studies of native plants as well as on a larger scale.

## Conclusions

Nowadays the search for natural, biologically active molecules is highly needed. Bioprospection of microorganisms adapted to extreme environmental conditions offers great opportunities for finding new biotechnological solutions. The selection of the Antarctic *Pseudomonas* sp. ANT_H4 strain that produce pyomelanin into the environment, as well as establishing its efficient overproduction using the fosmid expression system, made it possible considering the applicability of extracted biopolymer. Besides the basic, confirmed properties of ANT_H4-derived pyomelanin, such as sun and free radical protection and interaction with iron ions, we revealed its priming abilities to in vitro hairy root cultures. These interesting results requires further research on native plants in their natural environment and thus, subsequent studies will be directed into development of novel and sustainable agrotechnical solutions based on pyomelanin.

## Methods

### Bacterial strains, vectors, and culture conditions

*Pseudomonas* sp. ANT_H4 was obtained from a collection of bacterial cultures that were previously isolated from soil samples taken in 2012 from King George Island (Antarctica; GPS coordinates: 62 09.6010 S, 58 28.4640 W) [6]. *E. coli* MPO554 and pMPO579 vectors were used for fosmid library construction [41]. For subcloning, *E. coli* TG1 [62] along with plasmid pBluescript II SK [63] were used.

All strains were grown in LB medium in aerial conditions (3 RCF, Relative Centrifugal Force). *Pseudomonas* sp. ANT_H4 was grown at 18 °C while *E. coli* MPO554 and *E. coli* TG1 were grown at 37 °C. For the fosmid expression, the medium was supplemented with 12.5 µg/mL chloramphenicol, 800 µg/mL salicylate, 100 µg/mL arabinose and 1 mg/mL L-tyrosine. To obtain an expression of genes cloned in fosmid, it was supplemented with 50 μg/mL X-gal, 120 µg/mL IPTG, and 100 µg/mL ampicillin. When necessary, media were solidified by the addition of 1.5% (w/v) agar.

### Hairy roots cultures

*Calendula officinalis* L. hairy root line CC16 was obtained from cotyledon explants infected with *Agrobacterium rhizogenes* strain ATCC 15,834 according to a previously described procedure [64]. The transformation was confirmed by GUS (β-glucuronidase) reporter gene system in the histochemical assay and by PCR analysis of the *rolC* gene. The roots were cultivated in a liquid-modified Murashige and Skoog (MS) medium [65] with halved macronutrient concentrations (½ MS, at 25 °C, in the dark on a rotatory shaker at 1.5 RCF). Subcultures were performed every 3 weeks by transferring 2 cm pieces of the young, branched root to 100 mL of a fresh medium.

### Analysis of the production of siderophores

To determine the number of siderophores produced, bacteria were cultivated for 7 days in a GASN medium at 18 °C with rotary shaking set to 3 RCF. The initial optical density at 600 nm (OD_600_) was 0.1. After 7 days of cultivation, bacteria were centrifuged (4000 RCF for 5 min) and supernatants were added in a 1:1 ratio to the CAS reagent (CAS-HDTMA—chrome azurol S- hexadecyltrimethylammonium bromide) [66]. GASN medium was used as a negative control, while deferoxamine mesylate salt (Sigma-Aldrich, Saint Louis, MI, USA), at a concentration of 0.025 mM, was used as a positive control. All experiments were performed in triplicate. After an hour of incubation, the absorbance at 630 nm was measured using an automated microplate reader.

### Analysis of the production of biosurfactants

To determine the presence of biosurfactants, bacterial cultures were cultivated for 7 days in LB medium at 18 °C with rotary shaking set to 3 RCF in two variants: (i) with 1% (w/v) sunflower oil and (ii) without this supplementation. The initial optical density at 600 nm (OD_600_) was 0.1 and the controls were uninoculated variants. The experiments were performed in triplicate. After 7 days of cultivation, bacteria were centrifuged (4000 RCF for 5 min) and supernatants were tested through the ring method (du Nouy method) using a Kruss Tensiometer K20 (Kruss GmbH, Hamburg, Germany) [67].

### Draft genome sequencing

Genomic DNA of the *Pseudomonas* sp. ANT_H4 was isolated using the cetyltrimethylammonium bromide (CTAB)/lysozyme method [68]. Illumina TruSeq libraries for each strain were constructed following the manufacturer’s instructions. The genomic libraries were sequenced on an Illumina MiSeq instrument (using the v3 chemistry kit) (Illumina, San Diego, CA, United States) in the DNA Sequencing and Oligonucleotide Synthesis Laboratory (oligo.pl) at the Institute of Biochemistry and Biophysics, Polish Academy of Sciences, Warsaw. Raw reads were processed using fastp [69] version 0.19.5 with the following flags: -cut_by_quality3 –cut_window_size 10 – cut_mean_quality 25 –trim_poly_x –poly_x_min_len 15 – length_required 100. Filtered reads were used for assembly with SPAdes version 3.11.1 with **–**careful flag.

### Bioinformatic analysis

The analyzed bacterial genomes were automatically annotated using RAST [70] on the PATRIC 3.6.8 [71] web service and manually curated. Similarity searches were performed using BLAST programs [72]. The metabolic features were identified with the SEED viewer webserver [73], KEGG (Kyoto Encyclopedia of Genes and Genomes) Automatic Annotation System (KAAS) database [74] and the bacterial version of the antiSMASH webserver [75]. All options were selected with the default parameters. Additionally, for deeper metabolic investigation, the amino acid sequences were subjected to analysis with BLAST-KOALA [76]. KO (KEGG Orthology) assignments were performed using a modified version of the internally used KOALA (KEGG Orthology And Links Annotation) algorithm (BLASTKOALA), after a BLAST search against a non-redundant dataset of pangenome sequences [76]. Hits showing at least 50% identity with the reference protein were considered significant. Each hit was verified manually using BLASTp analysis.

The analysis of the spread and conservation of proteins encoded within the core of identified fosmids was performed throughout the protein search of proteins from within the core against the protein non-redundant (nr) National Center for Biotechnology Information (NCBI) database (accessed on April 14, 2022) with the application of diamond v2.0.13 using the following parameters: –ultra-sensitive –query-cover 75 –subject-cover 75 –id 75 –max-target-seqs 0 [77]. Based on the subject proteins’ accession numbers, the location of their genes was accessed with NCBI E-utilities. Only INSDC database-originating records were considered. The alignment of selected contigs was performed with clinker v0.0.20 with a 75% sequence identity threshold [78].

### Fosmid library preparation and DNA cloning

The ANT_H4 DNA of 30–50 kb was end-repaired to blunt end and cloned into the copy control pMPO579 vector using the CopyControl Fosmid Library Production Kit, according to the manufacturer's protocol [41, 79].

For subcloning, amplification of the relevant DNA region of the previously isolated ANT_H4-derived fosmid was performed by PCR using synthetic oligonucleotide primers, dNTPs and HiFi polymerase (Qiagen; with supplied buffer) in a Mastercycler (Eppendorf). The forward and reverse primers used in this study were 5′CGTACTGCAGGTCGTTACGGTTCATCTTGTG and 5′GACTCTCGAGTGTAATAGGCCGTTACCAGTC, respectively. The PCR product was then analyzed by electrophoresis on 0.8% agarose gel. The confirmed product of 1,689 bp was then digested with XhoI and PstI restriction enzymes and cloned into the multiple cloning site located within the *lacZ*α sequence of pBluescript II SK to facilitate selection by standard blue-white screening. Further transformation into *E. coli* TG1 was performed according to the Kushner method [62].

### Quantification of melanin-like compounds

The ANT_H4 strain was cultivated overnight in lysogeny broth (LB) medium at 18 °C with rotary shaking set to 3 RCF. Cultures were then centrifuged (4000 RCF for 5 min) and washed three times with 0.85% saline solution. Next, bacteria were added in triplicate to the fresh LB medium supplemented with 0.1% (w/v) L-tyrosine and cultivated at 18 °C with rotary shaking set to 3 RCF. The initial optical density at 600 nm (OD_600_) was 0.1. The idiophase was established after 5 days of cultivation (confirmed by the number of colony-forming units (CFU) counted after two consecutive days) and the experiment was continued up to day 10 to maximize metabolite production. Bacteria were then centrifuged (4000 RCF for 5 min) and the supernatant was measured spectrophotometrically at a wavelength of 400 nm [30]. A standard curve was prepared with polymerized homogentisic acid (Sigma-Aldrich) while the LB medium was a blank. Purification of the metabolite followed from the bacterial-free supernatant which was acidified with HCl solution to pH 2 and left overnight for melanin precipitation. In the next step, the residue was washed three times with ddH_2_O and ethanol. To reduce runaway esterification reactions, the metabolite was dried out of the alcohol. To dissolve the compound, the precipitate was suspended in ddH_2_O at pH 11 (NaOH solution). For the majority of experiments, the pH was adjusted to 7, which did not affect the solubility of the compounds. In order to remove the salt, the resuspended metabolite was then dialyzed in 10 kDa dialysis tubing for 24 h against water [15, 30].

### High-performance liquid chromatography (HPLC)

The purified and water-dissolved melanin-like compound from ANT_H4 was screened using an Agilent 1100 HPLC system (Agilent Technologies) with a DAD UV detector. Multiple wavelength monitoring was performed at λ = 216, 254, and 310 nm. 10 µl of the sample was injected onto an HPLC column (100 × 4.60 mm) filled with 5 µm Luna-100 C-18 (Phenomenex, Aschaffenburg, Germany). The samples were analyzed by linear gradient elution using H_2_O + 0.1% formic acid as solvent A and acetonitrile + 0.1% formic acid as solvent B at a flow rate of 2 mL/min. For solvent B, the gradient was from 5 to 100% in 10 min with a 3 min isocratic elution at 100% [80].

### Fourier-transform infrared (FT-IR) spectroscopy

Infrared spectra were recorded using the Nicolet iS50 FT-IR spectrometer (Thermo Scientific, Waltham, MA, United States) with a DTGS detector. Sample preparation was carried out as follows: 2 mg of dried melanin compound was mixed with 230 mg of KBr (Sigma-Aldrich) and carefully ground with agate mortar. Pellets were prepared using a press under a pressure of 10 T for 20 s. To collect spectra, the transmission mode was used with a resolution of 4 cm^−1^ and typically 256 scans were taken for each sample. A KBr pellet was used as a reference background [81].

### Free radical scavenging activity

The DPPH (2,2-diphenyl-1-picrylhydrazyl) method was used to measure the free radical scavenging activity. The dilutions were prepared as follows: 2 mL of 0.1 mM DPPH in methanol was added to 2 mL of methanol containing different amounts of melanin-like compound, i.e., to reach final concentrations of 1, 0.5, 0.25, 0.1, 0.01, and 0.001 mg/mL. The absorbance at 517 nm was measured spectrophotometrically (Evolution 260 Bio, Thermo Fisher Scientific) after 30 min. The scavenging of the DPPH radical (%) was calculated according to the formula ((A0 − A1)/A0 × 100) [82], where A0 is the absorbance of the control reaction and A1 is the absorbance of reactions containing melanin from the ANT_H4 strain. Each experiment was performed in triplicate.

### Sun protection factor (SPF) analysis

0.1 g of commercial sunscreen marked with an SPF of 5, 15, and 25, respectively, was added to 10 mL of absolute ethanol. To this solution, a melanin-like compound was added to final concentrations of 0.1 and 0.01 mg/mL. A variant without supplementation was used as a control. The absorbance of the mixture in the UV range (290–320 nm) was quantified at 5 nm intervals using ethanol as the blank. SPFs were calculated according to the Mansur method [83, 84]. Each experiment was performed in triplicate.

### Bioconsolidation assay

In order to establish the ability of the melanin-like compound to increase iron retention, 10 mL of two water solutions of melanin (0.1 and 0.01 mg/mL) were incubated on a mechanical cradle with 1 g of hematite (Geogut, Poland) and pyrite (Geogut) for 1, 24, and 48 h. The extracts were then filtered through 0.22-µm filters and submitted for further analysis. Distilled water was used as a control extraction solution. All extractions were performed in triplicate. The initial pH of the melanin solutions was 7.

### Analysis of iron concentration in the samples

The amount of iron was measured by Graphite Furnace Atomic Absorption Spectroscopy (GFAAS) using a Thermo Scientific SOLAAR M Series (TJA Solution, SOLAAR M, Cambridge, United Kingdom). The gas mixture was air and acetylene. The graphite cuvette duty cycle was as follows: evaporation—100 °C/30 s; incineration—1100 °C/20 s; atomization2,2-diphenyl-1-picrylhydrazyl 2100 °C/3 s; cleaning—2500 °C/3 s, and the calibration curve range was 0–20 µg/l with a lower limit of quantification of 0.1 µg/l. A deuterium lamp (TJA Solution) was used for background correction. Iron standard solutions (Merck, Darmstadt, Germany) were prepared in 3% HNO_3_.

### Iron chelation assay

In order to establish chelating properties, 0.01, 0.1, 0.25, 0.5, 1, 2, and 4 mg/mL of water solutions of pyomelanin were added in a 1:1 ratio to the CAS (chrome azurol S) reagent [66]. Distilled water was used as a negative control. The standard curve (R^2^ = 0.99) was prepared against deferoxamine mesylate salt (Sigma-Aldrich). All experiments were performed in triplicate. After an hour of incubation, the absorbance at 630 nm was measured using an automated microplate reader (Sunrise TECAN, Tecan Trading AG, Männedorf, Switzerland).

### In vitro hairy roots cultivation

*Calendula officinalis* L. hairy root cultures were produced in a ½ MS medium at a temperature of 24 °C, in the dark, on a shaker (1.5 RCF). Weighed samples of 3-week-old roots were transferred to a fresh medium, to which an aqueous solution of pyomelanin was added at a concentration of 0.01% (w/v). The control variant was distilled water. The tests were carried out in triplicate per variant after: (T1) transferring to a new medium with an elicitor, (T2) 7, and (T3) 14 days.

### Extraction of primary and secondary metabolites from hairy roots cultures

The hairy roots were weighed and extracted with: (i) diethyl ether for the determination of free sterols and oleanolic acid (pentacyclic triterpenoid) and (ii) methanol to investigate sterol glycosides and oleanolic acid saponins. The post-culture medium was also tested for the presence of isolated oleanolic acid saponins. The extraction of plant metabolites from hairy roots tissues was carried out in Soxhlet apparatuses. Saponins (extracted with butanol from post-culture medium as well as saponins from hairy root tissue) and sterol glycosides underwent acidic hydrolysis to release aglycones [60]. They were then fractionated against sitosterol and oleanolic acid (Sigma-Aldrich) standards using thin layer chromatography (TLC) in an appropriate solvent system (chloroform: methanol 97:3 v/v). Prior to analysis by gas chromatography with mass spectrometry (GC–MS), fractions containing oleanolic acid were additionally methylated [59].

### Identification and quantification of triterpenoids by gas chromatography coupled to a flame ionization detector and mass spectrometer (GC-FID/MS)

An Agilent Technologies 7890 A gas chromatograph equipped with a 5975C mass spectrometric detector was used for qualitative and quantitative analyses. Samples dissolved in diethyl ether:methanol (5:1, v/v) were applied (in a volume of 1–4 μL) using a 1:10 split injection. The column used was a 30 m × 0.25 mm (L × I.D), 0.25 μm particle size (HP-5MS UI, Agilent Technologies, Santa Clara, CA, USA). Helium was used as the carrier gas at a flow rate of 1 mL/min. The separation was made either under isothermal conditions at 280 °C or at the programmed temperature; an initial temperature of 160 °C held for 2 min, then increased to 280 °C at 5 °C/1 min, and the final temperature of 280 °C held for a further 44 min. Additional parameters were employed as follows: inlet and FID temperature 290 °C; MS transfer line temperature 275 °C; quadrupole temperature 150 °C; ion source temperature 230 °C; EI 70 eV; m/z range 33–500; FID gas (H2) flow 30 mL/min (hydrogen generator); and airflow 400 mL/min. Individual compounds were identified by comparing their mass spectra with library data from Wiley 9th ED. and NIST 2008 Lib. SW Version 2010 or previously reported data, and by comparison of their retention times and corresponding mass spectra with those of authentic standards, when available. Quantitation was performed using an external standard method based on calibration curves determined for the compounds belonging to representative triterpenoid classes; α-amyrin for triterpene alcohols, oleanolic acid methyl ester for triterpene acid methyl esters, and sitosterol for steroids.

### Statistical analysis

The significance of the differences between the mean values of the control and treated plant and soil samples was statistically evaluated by a two-tailed t-test at p ≤ 0.05. The Mann–Whitney–Wilcoxon test was applied whenever data failed to present a normal distribution or had different variances. The statistical analysis was carried out using the XLSTAT (version 2022.1) program.

## Supplementary Information

**Additional file 1: Table S1. **GC-MS data (retention times and characteristic ions of mass spectra) of identified steroids and triterpenoids. **Table S2. **The content of steroids and triterpenoid alcohols in hairy roots tissue. **Table S3. **The content of sterol glycosides in hairy roots tissue. **Table S4**. Free oleanolic acid (OA) content in hairy roots tissue. **Table S5**. Oleanolic acid saponins (OA) released to the medium. **Table S6**. Oleanolic acid saponins (OA) content in hairy roots tissue.

## Data Availability

The datasets used and/or analysed during the current study are available from the corresponding author on reasonable request.

## References

[1] Silva TR, Tavares RSN, Canela-Garayoa R, Eras J, Rodrigues MVN, Neri-Numa IA (2019). Chemical characterization and biotechnological applicability of pigments isolated from Antarctic bacteria. Mar Biotechnol.

[2] Ruiz B, Chávez A, Forero A, García-Huante Y, Romero A, Sánchez M (2010). Production of microbial secondary metabolites: regulation by the carbon source. Crit Rev Microbiol.

[3] Styczynski M, Biegniewski G, Decewicz P, Rewerski B, Debiec-Andrzejewska K, Dziewit L (2022). Application of psychrotolerant Antarctic bacteria and their metabolites as efficient plant growth promoting agents. Front Bioeng Biotechnol.

[4] Styczynski M, Rogowska A, Gieczewska K, Garstka M, Szakiel A, Dziewit L (2020). Genome-based insights into the production of carotenoids by Antarctic bacteria, *Planococcus* sp. ANT_H30 and *Rhodococcus* sp. ANT_H53B. Molecules.

[5] Silva TR, e., Silva LCF, de Queiroz AC, Alexandre Moreira MS, de Carvalho Fraga CA, de Menezes GCA, (2021). Pigments from Antarctic bacteria and their biotechnological applications. Crit Rev Biotechnol.

[6] Romaniuk K, Ciok A, Decewicz P, Uhrynowski W, Budzik K, Nieckarz M (2018). Insight into heavy metal resistome of soil psychrotolerant bacteria originating from King George Island (Antarctica). Polar Biol.

[7] Weimer A, Kohlstedt M, Volke DC, Nikel PI, Wittmann C (2020). Industrial biotechnology of *Pseudomonas* putida: advances and prospects. Appl Microbiol Biotechnol.

[8] Nawaz A, Chaudhary R, Shah Z, Dufossé L, Fouillaud M, Mukhtar H (2021). An overview on industrial and medical applications of bio-pigments synthesized by marine bacteria. Microorganisms.

[9] Pavan ME, López NI, Pettinari MJ (2020). Melanin biosynthesis in bacteria, regulation and production perspectives. Appl Microbiol Biotechnol.

[10] Turick CE, Knox AS, Becnel JM, Ekechukwu AA, Millike CE, Elnashar M (2010). Properties and function of pyomelanin. Biopolymers.

[11] Weidenfeld I, Zakian C, Duewell P, Chmyrov A, Klemm U, Aguirre J (2019). Homogentisic acid-derived pigment as a biocompatible label for optoacoustic imaging of macrophages. Nat Commun.

[12] Ben-David Y, Zlotnik E, Zander I, Yerushalmi G, Shoshani S, Banin E (2018). SawR a new regulator controlling pyomelanin synthesis in *Pseudomonas aeruginosa*. Microbiol Res.

[13] Hunter RC, Newman DK (2010). A putative ABC transporter, HatABCDE, is among molecular determinants of pyomelanin production in *Pseudomonas aeruginosa*. J Bacteriol.

[14] Bai R, Yu Y, Wang Q, Shen J, Yuan J, Fan X (2020). Laccase-catalyzed polymerization of hydroquinone incorporated with chitosan oligosaccharide for enzymatic coloration of cotton. Appl Biochem Biotechnol.

[15] Lorquin F, Ziarelli F, Amouric A, Di Giorgio C, Robin M, Piccerelle P (2021). Production and properties of non-cytotoxic pyomelanin by laccase and comparison to bacterial and synthetic pigments. Sci Rep.

[16] Pey AL, Megarity CF, Timson DJ (2019). Biosci Rep.

[17] AlKhatib M, Costa J, Spinelli D, Capecchi E, Saladino R, Baratto MC (2021). Homogentisic acid and gentisic acid biosynthesized pyomelanin mimics: Structural characterization and antioxidant activity. Int J Mol Sci.

[18] Zheng H, Chatfield CH, Liles MR, Cianciotto NP (2013). Secreted pyomelanin of *Legionella pneumophila* promotes bacterial iron uptake and growth under iron-limiting conditions. Infect Immun.

[19] Liu GY, Nizet V (2009). Color me bad: microbial pigments as virulence factors. Trends Microbiol.

[20] Abramovitch RB, Anderson JC, Martin GB (2006). Bacterial elicitation and evasion of plant innate immunity. Nat Rev Mol Cell Biol.

[21] Rogowska A, Szakiel A (2021). Enhancement of phytosterol and triterpenoid production in plant hairy root cultures—Simultaneous stimulation or competition?. Plants.

[22] Bolognese F, Scanferla C, Caruso E, Orlandi VT (2019). Bacterial melanin production by heterologous expression of 4-hydroxyphenylpyruvate dioxygenase from *Pseudomonas aeruginosa*. Int J Biol Macromol.

[23] Seo D, Choi KY (2020). Heterologous production of pyomelanin biopolymer using 4-hydroxyphenylpyruvate dioxygenase isolated from *Ralstonia pickettii* in *Escherichia coli*. Biochem Eng J.

[24] Hocquet D, Petitjean M, Rohmer L, Valot B, Kulasekara HD, Bedel E (2016). Pyomelanin-producing *Pseudomonas aeruginosa* selected during chronic infections have a large chromosomal deletion which confers resistance to pyocins. Environ Microbiol.

[25] Kurian NK, Bhat SG (2018). Data on the characterization of non-cytotoxic pyomelanin produced by marine *Pseudomonas stutzeri* BTCZ10 with cosmetological importance. Data Br.

[26] Mahmood HM, Mohammed AK, Flayyih MTA (2015). Purification and physiochemical characterization of pyomelanin pigment produced from local *Pseudomonas aeruginosa* isolates. World J Pharm Res.

[27] Cassaro A, Pacelli C, Baqué M, de Vera JPP, Böttger U, Botta L (2021). Fungal biomarkers stability in mars regolith analogues after simulated space and Mars-like conditions. J Fungi.

[28] Ma W, Waffo-Téguo P, Alessandra Paissoni M, Jourdes M, Teissedre PL (2018). New insight into the unresolved HPLC broad peak of *Cabernet Sauvignon* grape seed polymeric tannins by combining CPC and Q-ToF approaches. Food Chem.

[29] Miller KK, Springthorpe SK, Imbrogno J, Walker DJF, Gadiyar S, Keitz BK (2022). Biocompatible materials enabled by biobased production of pyomelanin isoforms using an engineered *Yarrowia lipolytica*. Adv Funct Mater.

[30] Zeng Z, Guo XP, Cai X, Wang P, Li B, Yang JL (2017). Pyomelanin from *Pseudoalteromonas lipolytica* reduces biofouling. Microb Biotechnol.

[31] Schmaler-Ripcke J, Sugareva V, Gebhardt P, Winkler R, Kniemeyer O, Heinekamp T (2009). Production of pyomelanin, a second type of melanin, via the tyrosine degradation pathway in *Aspergillus fumigatus*. Appl Environ Microbiol.

[32] Pralea IE, Moldovan RC, Petrache AM, Ilieș M, Hegheș SC, Ielciu I (2019). From extraction to advanced analytical methods: the challenges of melanin analysis. Int J Mol Sci.

[33] Medina-Salazar SA, Cornejo-Granados F, Equihua-Medina E, Ochoa-Leyva A, Vallejo-Pérez MR, Vega-Manriquez DX (2022). Genome analysis of *Pseudomonas* sp. 14A reveals metabolic capabilities to support epiphytic behavior. World J Microbiol Biotechnol.

[34] Chen C, Ali A, Su J, Wang Y, Huang T, Gao J (2021). *Pseudomonas stutzeri* GF2 augmented the denitrification of low carbon to nitrogen ratio: possibility for sewage wastewater treatment. Bioresour Technol.

[35] Xie F, Thiri M, Wang H (2021). Simultaneous heterotrophic nitrification and aerobic denitrification by a novel isolated *Pseudomonas mendocina* X49. Bioresour Technol.

[36] Perfumo A, Banat IM, Marchant R (2018). Going green and cold: biosurfactants from low-temperature environments to biotechnology applications. Trends Biotechnol.

[37] Tribelli PM, López NI (2018). Reporting key features in cold-adapted bacteria. Life.

[38] Jeoung JH, Bommer M, Lin TY, Dobbek H (2013). Visualizing the substrate-, superoxo-, alkylperoxo-, and product-bound states at the nonheme Fe(II) site of homogentisate dioxygenase. Proc Natl Acad Sci U S A.

[39] Ross D, Siegel D (2017). Functions of NQO1 in cellular protection and CoQ10 metabolism and its potential role as a redox sensitive molecular switch. Front Physiol.

[40] Zheng Q, Song Y, Zhang W, Shaw N, Zhou W, Rao Z (2015). Structural views of quinone oxidoreductase from *Mycobacterium tuberculosis* reveal large conformational changes induced by the co-factor. FEBS J.

[41] Terrón-González L, Medina C, Limón-Mortés MC, Santero E (2013). Heterologous viral expression systems in fosmid vectors increase the functional analysis potential of metagenomic libraries. Sci Rep.

[42] Lorquin F, Piccerelle P, Orneto C, Robin M, Lorquin J (2022). New insights and advances on pyomelanin production: from microbial synthesis to applications. J Ind Microbiol Biotechnol.

[43] Mesías LGG, Qwisgaard AMR, Untiveros GPC, Kobayashi LAP, Shimabukuro LEM, Sugahara AAK (2017). Comparison of the photoprotective effects of sunscreens using spectrophotometric measurements or the survivability of yeast cells exposed to UV radiation. Rev la Soc Química del Perú.

[44] Suryawanshi RK, Patil CD, Borase HP, Narkhede CP, Stevenson A, Hallsworth JE (2015). Towards an understanding of bacterial metabolites prodigiosin and violacein and their potential for use in commercial sunscreens. Int J Cosmet Sci.

[45] Ben Tahar I, Kus-Liśkiewicz M, Lara Y, Javaux E, Fickers P (2020). Characterization of a nontoxic pyomelanin pigment produced by the yeast *Yarrowia lipolytica*. Biotechnol Prog.

[46] Reis-Mansur MCPP, Cardoso-Rurr JS, Silva JVMA, de Souza GR, Cardoso V da S, Mansoldo FRP, et al. Carotenoids from UV-resistant Antarctic *Microbacterium* sp. LEMMJ01. Sci Rep. 2019;9:9554. 10.1038/s41598-019-45840-6 PMC6606617 31266976

[47] Rangel-Montoya EA, Paolinelli M, Rolshausen P, Hernandez-Martinez R (2020). The role of melanin in the grapevine trunk disease pathogen *Lasiodiplodia gilanensis*. Phytopathol Mediterr.

[48] Jiang C, Garg S, Waite TD (2015). Hydroquinone-mediated redox cycling of iron and concomitant oxidation of hydroquinone in oxic waters under acidic conditions: Comparison with iron-natural organic matter interactions. Environ Sci Technol.

[49] Chatfield CH, Cianciotto NP (2007). The secreted pyomelanin pigment of *Legionella pneumophila* confers ferric reductase activity. Infect Immun.

[50] Patel PR, Shaikh SS, Sayyed RZ (2018). Modified chrome azurol S method for detection and estimation of siderophores having affinity for metal ions other than iron. Environ Sustain.

[51] Abros’ Kin DP, Fuentes M, Garcia-Mina JM, Klyain OI, Senik SV, Volkov DS (2016). The effect of humic acids and their complexes with iron on the functional status of plants grown under iron deficiency. Eurasian Soil Sci.

[52] Bocanegra MP, Lobartini JC, Orioli GA (2006). Plant uptake of iron chelated by humic acids of different molecular weights. Commun Soil Sci Plant Anal.

[53] Mahmoud YAG (2004). Uptake of radionuclides by some fungi. Mycobiology.

[54] Hong L, Simon JD (2007). Current understanding of the binding sites, capacity, affinity, and biological significance of metals in melanin. J Phys Chem.

[55] Adjimani JP, Asare P (2015). Antioxidant and free radical scavenging activity of iron chelators. Toxicol Reports.

[56] Novellino L, Napolitano A, Prota G (1999). 5,6-Dihydroxyindoles in the fenton reaction: a model study of the role of melanin precursors in oxidative stress and hyperpigmentary processes. Chem Res Toxicol.

[57] Rogowska A, Szakiel A (2020). The role of sterols in plant response to abiotic stress. Phytochem Rev.

[58] González-Coloma A, López-Balboa C, Santana O, Reina M, Fraga BM (2011). Triterpene-based plant defenses Phytochem Rev.

[59] Rogowska A, Pączkowski C, Szakiel A (2022). Modulation of steroid and triterpenoid metabolism in *Calendula officinalis* plants and hairy root cultures exposed to cadmium stress. Int J Mol Sci.

[60] Alsoufi ASM, Staśkiewicz K, Markowski M (2021). Alterations in oleanolic acid and sterol content in marigold (*Calendula officinalis*) hairy root cultures in response to stimulation by selected phytohormones. Acta Physiol Plant.

[61] Alsoufi ASM, Pączkowski C, Szakiel A, Długosz M (2019). Effect of jasmonic acid and chitosan on triterpenoid production in *Calendula officinalis* hairy root cultures. Phytochem Lett.

[62] Kushner SR, Boyer HB, Nicosia S (1978). An improved method for transformation of *E. coli* with ColE1 derived plasmids. Genet Eng (N Y).

[63] Alting-Mees MA, Short JM (1989). pBluescript II: gene mapping vectors. Nucleic Acids Res.

[64] Długosz M, Wiktorowska E, Wiśniewska A, Paczkowski C (2013). Production of oleanolic acid glycosides by hairy root established cultures of *Calendula officinalis* L. Acta Biochim Pol.

[65] Murashige T, Folke S (1962). A revised medium for rapid growth and bio assays with tobacco tissue cultures. Physiol Plant.

[66] Schwyn B, Neilands JB (1987). Universal chemical assay for the detection and determination of siderophores. Anal Biochem.

[67] Malavenda R, Rizzo C, Michaud L, Gerçe B, Bruni V, Syldatk C (2015). Biosurfactant production by Arctic and Antarctic bacteria growing on hydrocarbons. Polar Biol.

[68] Sambrook J, Russell DW (2001). Molecular cloning: a laboratory manual III. Red.

[69] Chen S, Zhou Y, Chen Y, Gu J (2018). Fastp: an ultra-fast all-in-one FASTQ preprocessor. Bioinformatics.

[70] Aziz RK, Bartels D, Best AA, DeJongh M, Disz T, Edwards RA (2008). The RAST Server: rapid annotations using subsystems technology. BMC Genomics.

[71] Wattam AR, Davis JJ, Assaf R, Boisvert S, Brettin T, Bun C (2017). Improvements to PATRIC, the all-bacterial bioinformatics database and analysis resource center. Nucleic Acids Res.

[72] Altschul SF, Madden TL, Schaffer AA, Zhang J, Zhang Z, Miller W (1997). Gapped BLAST and PSI-BLAST: a new generation of protein database search programs. Nucleic Acids Res.

[73] Overbeek R, Olson R, Pusch GD, Olsen GJ, Davis JJ, Disz T (2014). The SEED and the rapid annotation of microbial genomes using subsystems technology (RAST). Nucleic Acids Res.

[74] Moriya Y, Itoh M, Okuda S, Yoshizawa AC, Kanehisa M (2007). KAAS: an automatic genome annotation and pathway reconstruction server. Nucleic Acids Res.

[75] Blin K, Shaw S, Kloosterman AM, Charlop-Powers Z, Van Wezel GP, Medema MH (2021). AntiSMASH 6.0: Improving cluster detection and comparison capabilities. Nucleic Acids Res.

[76] Kanehisa M, Sato Y, Morishima K (2016). BlastKOALA and GhostKOALA: KEGG tools for functional characterization of genome and metagenome sequences. J Mol Biol.

[77] Buchfink B, Reuter K, Drost H-G (2021). Sensitive protein alignments at tree-of-life scale using DIAMOND. Nat Methods.

[78] Gilchrist CLM, Chooi Y-H (2021). Clinker & clustermap.js: automatic generation of gene cluster comparison figures. Bioinformatics.

[79] Mizuno CM, Rodriguez-Valera F, Kimes NE, Ghai R (2013). Expanding the marine virosphere using metagenomics. PLoS Genet.

[80] Dang T, Loll B, Müller S, Skobalj R, Ebeling J, Bulatov T (2021). Molecular basis of antibiotic self-resistance in a bee larvae pathogen. BioRxiv.

[81] Kasztelan M, Słoniewska A, Gorzkowski M, Lewera A, Pałys B, Zoladek S (2021). Ammonia modified graphene oxide – gold nanoparticles composite as a substrate for surface enhanced Raman spectroscopy. Appl Surf Sci.

[82] Szakiel A, Voutquenne-Nazabadioko L, Henry M (2011). Isolation and biological activities of lyoniside from rhizomes and stems of *Vaccinium myrtillus*. Phytochem Lett.

[83] Dutra EA, Da Costa E, Oliveira DAG, Kedor-Hackmann ERM, Miritello Santoro MIR (2004). Determination of sun protection factor (SPF) of sunscreens by ultraviolet spectrophotometry. Rev Bras Ciencias Farm J Pharm Sci.

[84] de Pinho JJRG, de Freitas TS, Pinho DJMR, Alves MS, de Sousa OV. Determinação do fator de proteção solar (*in vitro*) de produtos magistrais na forma de gel, avaliação dos aspectos sensoriais e físico-químicos. HU Rev. 2014;81–8.

