# Supplementary material for: Heterologous production and characterization of a pyomelanin of Antarctic Pseudomonas sp. ANT_H4: a metabolite protecting against UV and free radicals, interacting with iron from minerals and exhibiting priming properties toward plant hairy roots
Source: Microb Cell Fact. 2022 Dec 16;21:261. doi: 10.1186/s12934-022-01990-3 (PMC9756463; doi:10.1186/s12934-022-01990-3)
Supplement: Supplementary file 1 — Additional file 1: Table S1. GC-MS data (retention times and characteristic ions of mass spectra) of identified steroids and triterpenoids. Table S2. The content of steroids and triterpenoid alcohols in hairy roots tissue. Table S3. The content of sterol glycosides in hairy roots tissue. Table S4. Free oleanolic acid (OA) content in hairy roots tissue. Table S5. Oleanolic acid saponins (OA) released to the medium. Table S6. Oleanolic acid saponins (OA) content in hairy roots tissue. [file 12934_2022_1990_MOESM1_ESM.pdf]

# – Additional file 1 –

Heterologous production and characterization of a pyomelanin of Antarctic *Pseudomonas* sp. ANT_H4 – a metabolite protecting against UV and free radicals, interacting with iron from minerals and exhibiting priming properties toward plant hairy roots

Michal Styczynski [1], Agata Rogowska [2], Christine Nyabayo [3], Przemyslaw Decewicz [1], Filip Romaniuk [1], Cezary Pączkowski [2], Anna Szakiel [2], Roderich Suessmuth [3], Lukasz Dziewit [1]*

[1] Department of Environmental Microbiology and Biotechnology, Institute of Microbiology, Faculty of Biology, University of Warsaw, Warsaw, Poland
[2] Department of Plant Biochemistry, Institute of Biochemistry, Faculty of Biology, University of Warsaw, Warsaw, Poland
[3] Institute of Chemistry, Technical University of Berlin, Berlin, Germany

*** Corresponding Author: Lukasz Dziewit, phone: +48225541403; mail: l.dziewit@uw.edu.pl**

Running title: Pyomelanin of Antarctic *Pseudomonas* sp. ANT_H4

Keywords: Antarctica, biopolymer, *Pseudomonas*, priming of hairy roots, pyomelanin

Table S1. GC-MS data (retention times and characteristic ions of mass spectra) of identified steroids and triterpenoids

**Compound**	**Formula**	**Molecular weight**	**Retention time [min]**	**Mass spectrum** *m/z* **(relative intensity)**
cholesterol	$C_{27}H_{46}O$	386.6	31.06	386 (26), 107 (50), 105 (48), 91 (57), 81 (54), 79 (46), 69 (47), 57 (87), 55 (73), 43 (100), 41 (55)
campesterol	$C_{28}H_{48}O$	400.6	33.59	400 (30), 107 (51), 105 (55), 95 (49), 83 (45), 81 (64), 71 (62), 57 (77), 55 (77), 43 (100), 41 (52)
stigmasterol	$C_{29}H_{48}O$	412.6	34.52	412 (36), 145 (64), 107 (52), 95 (100), 83 (66), 81 (90), 78 (60), 69 (67), 67 (85), 55 (69)
sitosterol	$C_{29}H_{50}O$	414.7	36.15	414 (29), 145 (54), 107 (59), 105 (60), 95 (54), 91 (49), 81 (57), 57 (68), 55 (70), 43 (100)
isofucosterol	$C_{29}H_{48}O$	412.3	36.78	412 (5), 314 (100), 105 (47), 95 (50), 91 (42), 83 (40), 81 (51), 69 (61), 55 (96), 43 (49)
β-amyrin	$C_{30}H_{50}O$	426.7	37.13	426 (27), 219 (18), 218 (100), 203 (49), 189 (17), 135 (11), 109 (13), 105 (12), 95 (15), 81 (18), 69 (14)
α-amyrin	$C_{30}H_{50}O$	426.7	38.62	426 (4), 219 (18), 218 (100), 203 (20), 189 (19), 135 (17), 133 (15), 122 (16), 119 (15), 95 (16)
tremulone	$C_{29}H_{46}O$	410.7	39.20	410 (32), 187 (27), 174 (100), 161 (37), 159 (26), 91 (28), 57 (28), 55 (37), 43 (44), 41 (28)
24-methylenecycloartanol	$C_{30}H_{50}O$	426.7	40.50	440 (5), 121 (60), 119 (55), 109 (62), 107 (76), 105 (57), 95 (98), 93 (64), 81 (72), 69 (99), 55 (100)
oleanolic acid methyl ester	$C_{31}H_{50}O_3$	470.1	46.37	470 (1), 262 (48), 207 (13), 204 (16), 203 (100), 202 (21), 189 (22), 133 (17), 119 (13), 105 (14)

Table S2. The content of steroids and triterpenoid alcohols in hairy roots tissue

**Compound**	**Content [µg/g DW ± SD]**			
	**days**			
	7		14	
	C	E	C	E
cholesterol	19.19±3.64	14.69±1.16	30.31±0.78	43.12±1.87
campesterol	126.80±10.05	231.32±5.96	160.69±6.98	190.97±8.29
stigmasterol	848.76±67.30	1284.40±33.10	1342.80±58.29	1609.26±69.86
sitosterol	492.72±39.07	799.88±20.61	309.85±13.45	461.86±20.05
isofucosterol	251.18±19.92	412.29±10.63	299.95±13.02	348.98±15.15
β-amyrin	94.31±7.48	90.02±2.32	25.15±1.09	123.53±5.36
α-amyrin	85.21±6.76	135.32±3.49	280.63±12.18	361.45±15.69
tremulone	163.86±12.99	106.80±2.75	173.77±7.54	380.70±16.53
24-methylene-cycloartanol	72.39±5.74	118.85±3.06	104.84±4.55	127.14±5.52
**Total**	**2154.41**	**3193.56**	**2727.99**	**3647.00**

Table S3. The content of sterol glycosides in hairy roots tissue

**Compound**	**Content [µg/g DW ± SD]**			
	**days**			
	7		14	
	C	E	C	E
cholesterol	66.86±14.27	50.61±10.80	68.59±14.64	62.99±13.44
campesterol	75.02±16.01	89.11±19.01	128.20±27.35	121.47±25.92
stigmasterol	59.24±12.64	73.47±15.68	130.15±27.77	81.75±17.44
sitosterol	55.44±11.83	75.97±16.21	67.37±14.38	58.18±12.41
**Total**	**256.55**	**289.16**	**394.31**	**324.39**

Table S4. Free oleanolic acid (OA) content in hairy roots tissue

**Compound**	**Content [µg/g DW ± SD]**			
	**days**			
	7		14	
	C	E	C	E
OA	87.03±18.57	1616.40±307.08	176.25±37.61	16037.34±3046.72

Table S5. Oleanolic acid saponins (OA) released to the medium

**Compound**	**Content [mg/L×g DW ± SD]**			
	**days**			
	7		14	
	C	E	C	E
OA	1.45±0.27	1.98±0.38	2.38±0.45	2.91±0.69

Table S6. Oleanolic acid saponins (OA) content in hairy roots tissue.

**Compound**	**Content [µg/g DW ± SD]**			
	**days**			
	7		14	
	C	E	C	E
OA	313.30±66.85	116.27±24.81	409.77±87.44	139.82±29.83
